# Evaluation of Antimicrobial, Antiadhesive and Co-Aggregation Activity of a Multi-Strain Probiotic Composition against Different Urogenital Pathogens

**DOI:** 10.3390/ijms24021323

**Published:** 2023-01-10

**Authors:** Patrizia Malfa, Laura Brambilla, Silvana Giardina, Martina Masciarelli, Diletta Francesca Squarzanti, Federica Carlomagno, Marisa Meloni

**Affiliations:** 1 SynBalance srl, 21040 Origgio, VA, Italy; 2 VitroScreen srl, 20149 Milan, MI, Italy; 3 Complife Italia srl, 20024 Garbagnate Milanese, MI, Italy; 4 Roelmi HPC srl, 21040 Origgio, VA, Italy

**Keywords:** urogenital microbiota, probiotics, pathogens, infection, antimicrobial activity, co-aggregation

## Abstract

The urogenital microbiota is dominated by *Lactobacillus* that, together with *Bifidobacterium*, creates a physiological barrier counteracting pathogen infections. The aim of this study was to evaluate the efficacy of a multi-strain probiotic formulation (*Lactiplantibacillus plantarum* PBS067, *Lacticaseibacillus rhamnosus* LRH020, and *Bifidobacterium animalis* subsp. *lactis* BL050) to inhibit adhesion and growth of urogenital pathogens. The antimicrobial and antiadhesive properties of the probiotic strains and their mixture were evaluated on human vaginal epithelium infected with *Candida glabrata*, *Neisseria gonorrheae*, *Trichomonas vaginalis*, and *Escherichia coli*-infected human bladder epithelium. The epithelial tissue permeability and integrity were assessed by transepithelial/transendothelial electrical resistance (TEER). Co-aggregation between probiotics and vaginal pathogens was also investigated to elucidate a possible mechanism of action. The multi-strain formulation showed a full inhibition of *T. vaginalis*, and a reduction in *C. glabrata* and *N. gonorrheae* growth. A relevant antimicrobial activity was observed for each single strain against *E. coli*. TEER results demonstrated that none of the strains have negatively impaired the integrity of the 3D tissues. All the probiotics and their mixture were able to form aggregates with the tested pathogens. The study demonstrated that the three strains and their mixture are effective to prevent urogenital infections.

## 1. Introduction

Vaginal microbiota (VMB) is generally defined as a complex association of heterogeneous microorganisms which can influence the vaginal microenvironment. Indeed, the close interaction among these microorganisms can positively or negatively affect the host through mechanisms of commensalism, mutualism, and pathogenicity [1]. VMB composition and structure has been largely studied and it is widely acknowledged that it is subjected to important physiological modifications throughout the lifespan of women, from birth to menopause. Such changes can be correlated to different glycogen concentrations in the vagina, hormones levels, and the consequent pH alteration [2].

In the vagina, interactions between its microbiota and the human host represent the first line of defence against opportunistic pathogens. In normal conditions, a balanced VMB composition is considered in eubiosis. When this equilibrium is disrupted, opportunistic pathogens can prevail, leading to dysbiosis, a state of alteration of normal functionality that involves innate and immune-mediated responses, often leading to chronic inflammation [3], such as urinary tract infections (UTIs), common clinical conditions in which VMB plays an important role [4].

Generally, an eubiotic VMB is characterized by the dominant presence of different *Lactobacillus* species [3]. They produce lactic acid and thus are defined as lactic acid bacteria (LAB). *Lactobacillus* are responsible for the acidic environment of the vagina which, along with other mechanisms such as antimicrobial substances secretion and competitive exclusion, prevents pathogen growth in the urogenital tract. Lactic acid is a potent bactericidal compound since it causes the pH lowering, thus inhibiting most of the pathogens [5]. It is normally present in the vaginal environment in two racemic forms, D (−) and L (+) lactic acid. Such isomers, produced in different ratios depending on resident *Lactobacillus* spp., have shown specific inhibition effect against opportunistic microorganisms [3,5]. Previous studies stated that VMB with a predominance of *Lactobacillus crispatus* is characterized by higher levels of D (−) lactic acid, while L (+) lactic acid has specific immunological properties. Therefore, both isomers play an important role in the maintenance of a healthy vaginal environment [6]. Additionally, *Lactobacillus* are known to produce bacteriocins with inhibitory capacity against pathogens. These substances are small peptides, classified according to their molecular size, mode of action, presence of modified amino acids, and morphological traits [7,8]. Different studies on several *Lactobacillus* strains demonstrated their ability to produce a broad spectrum of antimicrobial bacteriocins, such as plantaricin, reuterin, and nisin [7]. Finally, *Lactobacillus* could block urogenital pathogen adhesion to mucosa epithelial cells throughout different mechanisms, such as exclusion, competition, or displacement [9]. During the exclusion process, LAB are able to colonize the epithelium and to occupy the binding sites, leaving no free surface for urological pathogen adhesion. Competition occurs instead when LAB and pathogens compete for nutrients in the environment, while displacement indicates microorganisms’ ability to remove pathogens from epithelial cells [10]. For all these reasons, a microbiota composition particularly enriched in *Lactobacillus* plays a pivotal role in the maintenance of eubiosis [11]. When an unbalanced situation occurs, their presence is reduced, and the consequent increase in pH causes overgrowth of undesirable or pathogenic microorganisms [12].

UTIs are frequent disorders characterized by pathogenic colonization of vagina, urethra, and bladder, which sometimes reaches kidneys, causing infection [4]. The proximity of these districts allows a gradual cross-contamination which can lead to more severe infections [13]. *Escherichia coli*, *Atopobium vaginae*, *Gardnerella vaginalis*, *Klebsiella pneumoniae*, *Proteus mirabilis*, *Enterococcus faecalis*, and *Staphylococcus saprophyticus* species have been recognized as the most common responsible for urinary tract diseases [14,15]. In particular, the Gram-negative bacterium *E. coli* is able to produce several soluble metabolic products with potential tissue-damaging effects, such as pore forming toxins and proteases. Its adherence ability is mediated by slime production, which confer to *E. coli* higher virulence, higher resistance to the phagocytosis, prevention of antimicrobial substances access, and improved adherence to host tissues. Moreover, *E. coli* along with *Candida* spp. are considered as main etiological agents of common diseases in the vagina [16].

Antimicrobials compounds such as fluconazole, metronidazole and clindamycin are used as common therapy for urogenital infections [17,18]. Nevertheless, long-term antimicrobial drug administration is reported to be related to antibiotic resistance and consequent recurrences [19,20]. In the last years, probiotic use for the treatment and prevention of vaginal infections has considerably increased [21]. Indeed, probiotics are reported not only to restore normal vaginal homeostasis by promoting the proliferation of beneficial microorganisms but also to reduce the associated symptoms (pain, inflammation, discomfort, etc.) [22,23]. *Lactobacillus*-based probiotics are also known to be particularly effective in counteracting urogenital pathologies [24]. Furthermore, some studies suggest the co-aggregation of *Lactobacillus* with pathogens as one of the mechanisms of action in the intestinal district [25,26].

The aim of this research was to determine the antimicrobial and co-adhesion activity of a multi-strain probiotic composition containing *Lactiplantibacillus plantarum* PBS067, *Lacticaseibacillus rhamnosus* LRH020, and *Bifidobacterium animalis* subsp. *lactis* BL050 against some common urogenital pathogens. This formulation (SynBalance^®^ Femme) was previously characterized for its probiotic properties both as single strains [27] and as their mixture [28,29]. In this work, further evaluations were performed on 3D reconstructed human vaginal epithelium (HVE) and human bladder epithelium (HBE). Moreover, in order to more deeply investigate the mechanism of action of SynBalance^®^ Femme on pathogens, its interaction as mixture and single strains, and possible co-aggregation with *Candida albicans*, *G. vaginalis*, and *E. coli* were also studied at the ultrastructural level by Scanning Electron Microscope (SEM) analysis, demonstrating the efficacy of the probiotic treatment.

## 2. Results

### 2.1. Antimicrobial and Antiadhesive Efficacy on HVE

HVE tissues were infected in vitro with three pathogenic strains: *Candida glabrata*, *Neisseria gonhorreae*, and *Trichomonas vaginalis*.

In the antimicrobial efficacy protocol, the probiotic formulation was applied on HVE after being colonized by the different pathogens. In all cases, viability and integrity of the tissues and probiotic concentration were not altered at the end of the experiment (Table 1).

*C. glabrata* colonized the tissues with an initial mean count of 7.75 log_10_ CFU/mL. After exposure to probiotic, an amount of 7.75 log_10_ and 5.43 log_10_ CFU/mL was found in tissue washing wastes and tissue homogenates, respectively. In the case of *N. gonhorreae*, the initial mean count was 7.81 log_10_ CFU/mL. No viable microorganisms were found in tissue washing wastes, while an amount of 4.69 log_10_ CFU/mL was found in tissue homogenates. *T. vaginalis* colonized the tissue with an initial mean count of 7.49 log_10_ CFU/mL. In this case, no viable microorganisms were found neither in the tissue washing wastes nor in the tissue homogenates (Table 1).

To evaluate the preventive-antiadhesive activity (Protocol B), SynBalance^®^ Femme (7.74 log10 CFU/mL initial count) was applied directly on HVE; after incubation, an inoculum of the three pathogenic microorganisms was carried out (Table 2).

As for Protocol A, the amount of probiotics was not affected by infection of pathogens. *C. glabrata* was applied with an initial mean concentration of 7.75 log_10_ CFU/mL. At the end of the experiment, the fungal concentration found in the tissue washing waste was 6.17 log_10_ CFU/mL, while no viable microorganisms were found in the tissue homogenates. Likewise, starting from 7.81 log10 CFU/mL of *N. gonhorreae*, the anti-adhesion activity was evaluated. No viable microorganisms were found in tissue washing waste and in the tissue media, while in tissue homogenates a count of 5.14 log_10_ CFU/mL was found. HVE, pretreated with the probiotic formulation, was also tested for *T. vaginalis* adhesion. Starting from an initial amount of 7.49 log_10_ CFU/mL, only 2.17 log_10_ CFU/mL and 3.48 log_10_ CFU/mL in tissue washing waste and in tissue homogenates were detected, respectively. In summary, on HVE the amount of viable pathogens was significantly reduced. Moreover, no significant changes in the epithelium viability and integrity was observed among treatments (Table 2).

### 2.2. Antibacterial and Antiadhesive Efficacy on HBE

In the antibacterial efficacy test (Protocol A), each strain of the probiotic mixture was applied on HBE, already colonized by *E. coli*. After incubation, the concentration of viable probiotics and pathogen was counted both in apical compartment and homogenate tissue. *B. animalis* subsp. *lactis* BL050 totally inhibited *E. coli* non-adherent cells (apical compartment) and significantly reduced the viable bacteria in the homogenate tissue by 1 log_10_, indicating antibacterial activity on *E. coli* cells. Both *L. plantarum* PBS067 and *L. rhamnosus* LRH020 showed an optimal performance, with a significant reduction in non-adherent cells and total depletion of *E. coli* cells in the homogenate tissues (Table 3, left part).

In order to evaluate the preventive-antiadhesive activity (Protocol B), the three probiotic strains were applied on HBE separately; after incubation, the tissue was inoculated with *E. coli*. For *B. animalis* subsp. *lactis* BL050 and *L. plantarum* PBS067 the reduction in viable *E. coli* cells in the tissue homogenates was less evident than in *L. rhamnosus*, while no decrease in non-adherent *E. coli* cells was found. For *L. rhamnosus* LRH020, 1 log_10_ reduction in non-adherent *E. coli* was observed in the apical compartment, while a total inhibition of *E. coli* cell growth was obtained in the HBE homogenates (Table 3, right part).

In parallel, the overall resistance of the tissue linked both to its thickness and to the integrity of tight junctions was assessed by TEER measurements. In Protocol A, TEER values, registered after 4 h of colonization with *E. coli* and 16 h treatment with the probiotic strains, did not shown any modification of the tissue barrier function compared to the negative control. As observed in Protocol A, no differences in the TEER values were registered also in Protocol B (Table 4).

**Figure 1:**
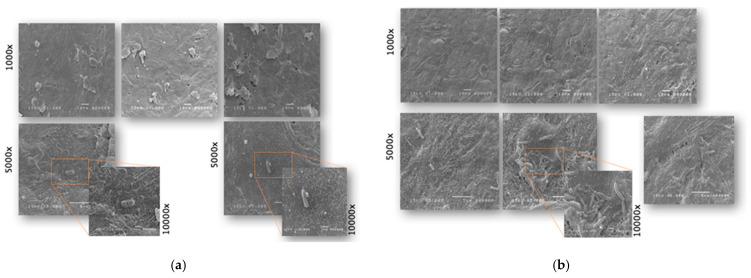
Representative SEM images of HBE tissue (**a**) colonized by *E. coli* (control) and (**b**) pretreated with *L. rhamnosus* LRH020. SEM image magnifications are reported near the figures.

The ultrastructural analysis by SEM showed the bacterial phenotype’s, defined as density, ability to adhere to the epithelium and to form biofilms. The HBE tissue colonized by *E. coli* showed a mild level of dryness. *E. coli* was strongly attached to the tissue and kept adherent to the surface by mucus (Figure 1a).

Pretreatment with *L. rhamnosus* LRH020 on HBE counteracted the observed *E. coli*-induced damages related to hydration, as demonstrated by a greater and more regular distribution of microvilli on the surface (Figure 1b) than in the control (*E. coli* only).

LAB were clearly visible on the surface, adhering closely to the HBE surface due to the production of extracellular matrix that allowed their aggregation and colonization. *E. coli* was not detected on the surface (Table 5).

### 2.3. Probiotic Microorganisms and Vaginal Pathogens Co-Aggregation

After mixing SynBalance^®^ Femme with the three different pathogens, *G. vaginalis*, *E. coli*, and *C. albicans*, flocculation times were recorded and clustered in four different groups of response: less than 15 min, 15–30 min, more than 30 min, and no evidence of precipitate (Table 6).

All precipitates were collected and fixed for SEM analysis. Magnifications were selected based on pathogen morphology and dimensions to highlight the formation of aggregates within the precipitate.

At 20,000×, *G. vaginalis* morphology showed a round shape of about 1 µm of diameter, while, at 10,000× *C. albicans* seemed organized in characteristic clusters formed by ovoidal cells with 3–5 µm of diameter. At 40,000×, *E. coli* exhibited its typical rod shape of 1.8 µm long and wide 583 nm.

**Figure 2:**
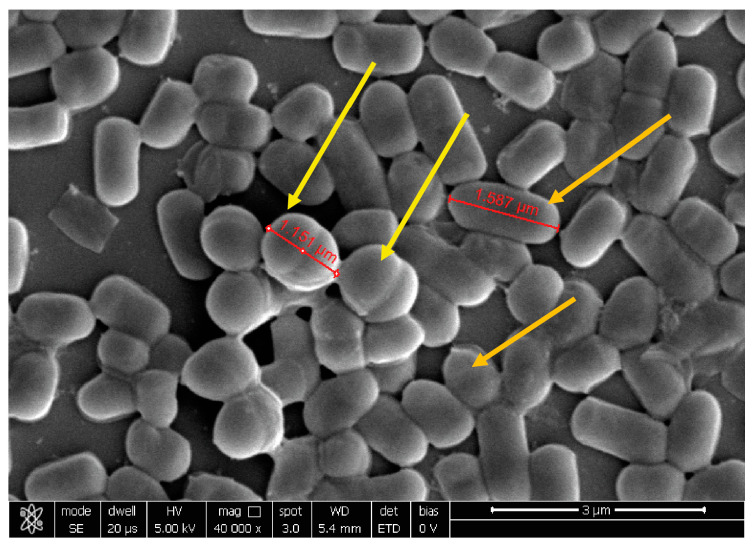
Representative image of *L. plantarum* PBS067 and *G. vaginalis* observed at SEM. The yellow arrows indicate *G. vaginalis*, the orange arrows *L. plantarum* PBS067. Magnification 40,000×.

Co-aggregation with *G. vaginalis* was well visible for *B. animalis* subsp. *lactis* BL050 and *L. plantarum* PBS067 (Figure 2), highlighting a dense carpet where pathogen and probiotic bacteria interplayed, while for *L. rhamnosus* LRH020 the two different population are organized individually.

**Figure 3:**
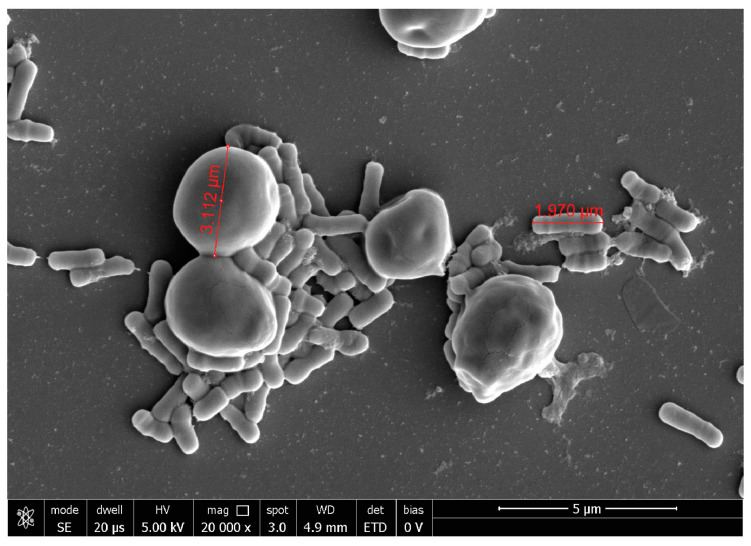
Representative image of *L. rhamnosus* LRH020 and *C. albicans* observed at SEM. Magnification 20,000×.

Due to the different morphology and size, the co-aggregation with *C. albicans* was very clear both for the single strains and their mixture. *L. rhamnosus* LRH020 was able to surround isolated *C. albicans* cells forming partial co-aggregates (Figure 3).

**Figure 4:**
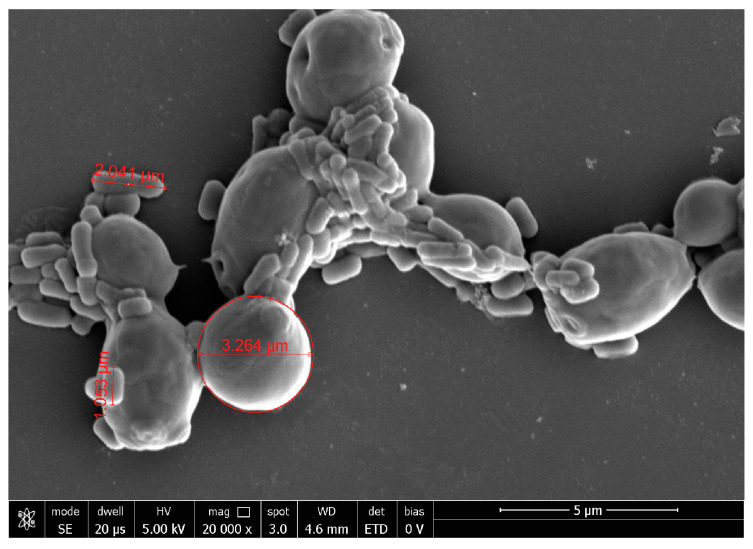
Representative image of SynBalance^®^ Femme mixture and *C. albicans* observed at SEM. Magnification 20,000×.

*B. animalis* subsp. *lactis* BL050 and *L. plantarum* PBS067 formed well visible clusters; similarly, a complete co-aggregation was observed for SynBalance^®^ Femme: fungal cells resulted embedded into the probiotic biofilm (Figure 4).

**Figure 5:**
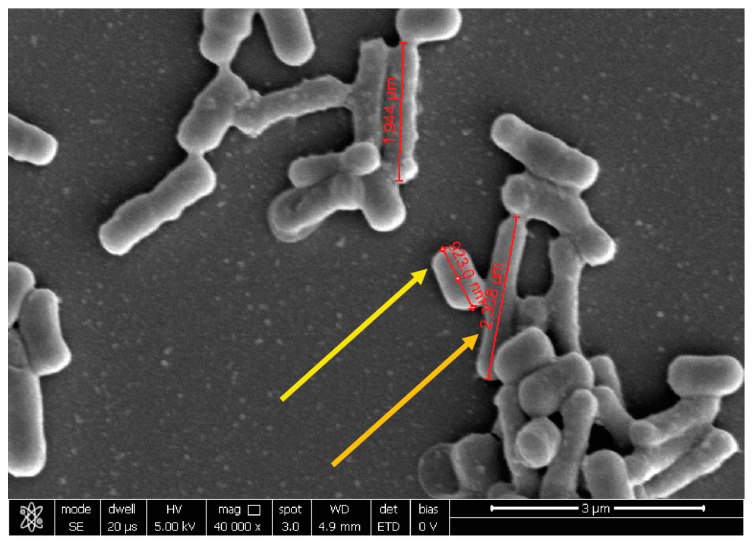
Representative image obtained with SEM of SynBalance^®^ Femme mixture and *E. coli*. The yellow arrow indicates the pathogen cell, while the orange arrow a probiotic bacterium. Magnification 40,000×.

The assessment of co-aggregation against *E. coli* was very difficult. For all the tested probiotic strains, the dense organization of the precipitate was evident but, due to the similar shape, the two populations are not clearly distinguishable. In the suspension of probiotic formulation and *E. coli*, the precipitates interact closely but it was difficult to appraise the distinct bacteria (Figure 5).

**Figure 6:**
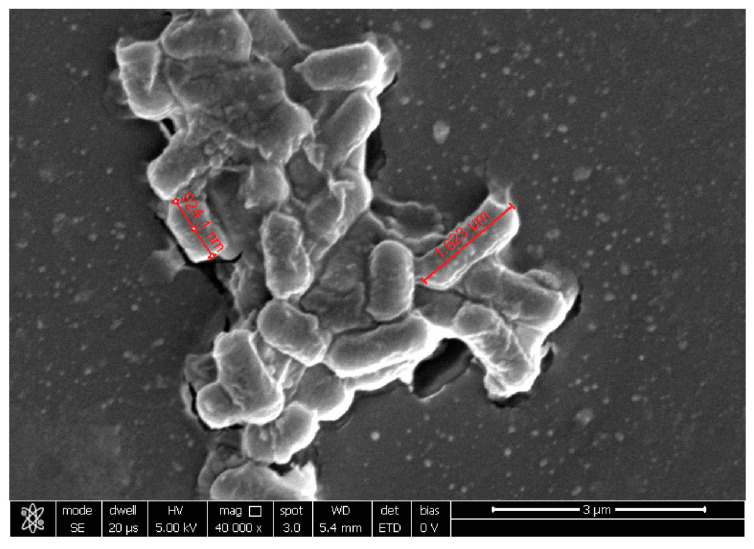
Representative image obtained with SEM of *L. plantarum* PBS067 and *E. coli* aggregates. Magnification 40,000×.

In the case of *L. plantarum* PBS067, however, the presence of a solid aggregate (Figure 6) was evident.

## 3. Discussion

The maintenance of the VMB equilibrium has a pivotal role in keeping a eubiotic and wholesome status [30]. The prevalence of good bacteria, in particular belonging to *Lactobacillus* genus, exerts an antimicrobial activity which inhibits the growth of different pathogenic microorganisms. When this balance is lost, the use of probiotics to restore a healthy microbiota is reported to be effective as an adjutant or alternative to antibiotic treatment [31]. Many studies on antimicrobial ability of probiotic bacteria have been carried out demonstrating that the efficacy against different pathogenic microorganisms is strain-specific [32]. Many authors have reported that urogenital infections can originate from lurked reservoirs present in the gut [33,34], confirming that an unhealthy gut microbiota can be responsible for infections such as vaginitis, cystitis, and pyelonephritis, due to the crosstalk between vaginal and intestinal district [35]. This connection is fundamental to understand the pathogenesis of UTIs and, consequently, to design a correct protocol to prevent them.

In this study, three probiotic strains and their mixture were investigated for their antimicrobial effects against different pathogens that cause urogenital infections.

Previously, the same probiotic formulation had already been extensively studied both in vitro for its antimicrobial activity and in clinical trials for several applications. In a randomized placebo-controlled pilot study, Mezzasalma et al. showed that the formulation possessed antimicrobial activity against both *E. coli* and *C. albicans*, and determined an enhancement in the amount of the specific strains due to its persistence in the vaginal microbiota after a wash out period of seven days [29]. Furthermore, the oral administration of SynBalance^®^ Femme formulation in women with recurrent bacterial vaginosis drove to a *Lactobacillus*-dominated vaginal microbiota, reducing dysbiosis condition and recurrence rate of bacterial vaginosis (BV) in the active group (16%) compared to the control group (40%) [36].

In order to understand the mechanisms of action of the probiotics tested and their mixture, two different protocols were applied to explore their antimicrobial efficacy, and preventive and anti-adhesive activity on vaginal and bladder reconstructed epithelia. These two different approaches were designed to mimic acute or chronic infections and to verify the effectiveness of tested probiotics in such conditions.

The results obtained in the experiment on HVE showed that only a part of *C. glabrata* remained attached to the epithelium after the addition of probiotic formulation to the system; a similar mechanism was observed for *N. gonorrhoeae*, demonstrating for these two pathogens the antimicrobial activity of the probiotic formulation tested. Different behavior was observed for *T. vaginalis*, where the pathogen has not been found in its viable form neither in the homogenate nor in the tissue washing phase; a full antimicrobial activity was registered, demonstrating the efficacy of SynBalance^®^ Femme for both adherent and non-adherent cells. Therefore, only a fraction of the initial pathogenic microorganism concentration remained attached to the epithelium treated with probiotics, showing a remarkable antimicrobial and anti-adhesion activity of SynBalance^®^ Femme.

A similar performance was observed on the bladder epithelia for each strain of the probiotic formulation, suggesting an antibacterial activity and the ability to counteract *E. coli* adhesion, both on apical compartment and in the tissue homogenates.

*B. animalis* supsp. *lactis* BL050 showed a strong antibacterial ability when applied on the established *E. coli*-colonized model, suggesting that this strain exerts its activity in the first hours of treatment. In the preventive model, the lack of activity could be explained as possible sensitivity to the tissue cultivation conditions, losing its efficacy after 16 h of incubation.

*L. plantarum* PBS067 showed a high efficacy in counteracting *E. coli* adhesion by detaching it from the HBE when it has already colonized the mucosa. The mechanism of action may involve the displacement of *E. coli* from the epithelial surface by competition with the same adhesion sites on the mucosa or by interference with the slime [37]. This ability was partially lost when this strain was applied as pretreatment. This outcome could be caused by a higher sensitivity of this probiotic to the tissue cultivation conditions, as observed for *B. lactis* BL050, thus limiting its efficacy against *E. coli*. However, a competition mechanism in the adhesion process of *E. coli* by displacement of the pathogenic bacterium is evident. On the contrary, *L. rhamnosus* LRH020 determined a total inhibition of *E. coli* adhesion when applied as preventive and antimicrobial agent. The absence of *E. coli* on the surface of HBE confirmed the efficacy of *L. rhamnosus* LRH020 in displacing *E. coli* after colonization. This event was also confirmed by SEM analysis, where a high colonization capacity by *L. rhamnosus* LRH020 was observed, with relative eradication of *E. coli*. A preservation of the microvilli structure was also observed, which are useful for the physiological role they play in preventing *E. coli*-induced tissue drying [38].

Another possible mechanism of action for the antagonistic activity against pathogen could be co-aggregation, i.e., the microorganisms’ ability to cluster together, forming stable multi-cellular associations. This phenomenon has been observed for the first time in human oral bacteria and it can occur among different genera and species [39]. It has been suggested that cellular aggregation could promote the colonization of beneficial microorganisms on human tissues, such as the intestinal and the vaginal tract, and it has been reported both for *Lactobacillus* and *Bifidobacterium* genera [40,41,42]. Co-aggregation ability represents a barrier to prevent surface colonization by pathogenic microorganisms [43,44]. In our study, the probiotic strains and their mixture showed a high capability to co-aggregate with different vaginal pathogens, suggesting that this property could allow them to survive at sufficiently high number and colonize the urogenital tract. The ability to co-aggregate with pathogens and to adhere to the epithelial cell surface is probably due to the presence of specific molecules involved in the mechanisms of binding microorganisms or cells [45]. This characteristic is not related only to the genera or species but it is a strain-dependent mechanism [25].

## 4. Materials and Methods

### 4.1. Bacterial Strains

*Lactiplantibacillus plantarum* PBS067 (DSM 24937), *Lacticaseibacillus rhamnosus* LRH020 (DSM 25568) and *Bifidobacterium animalis* subsp. *lactis* BL050 (DSM 25566) freeze-dried probiotic powders and their mixture called SynBalance^®^ Femme were provided by Roelmi HPC Srl (Origgio, VA, Italy). *Lactobacillus* were grown in homofermentative-heterofermentative differential (HHD) medium, while HHD supplemented with 0.3 g/L L-cysteine hydrochloride monohydrate (cys-HHD) medium was used for *Bifidobacterium* and SynBalance^®^ Femme formulation. The cultures were incubated at 37 °C under anaerobic conditions.

As antagonistic microorganisms, *Escherichia coli* (ATCC 8739), *Gardnerella vaginalis* (ATCC 14018), *Neisseria gonhorreae* (ATCC 43069), *Trichomonas vaginalis* (ATCC 30238), *Candida glabrata* (ATCC 15126), and *Candida albicans* (ATCC 10231) were investigated. *E. coli* was cultivated in Tryptic Soy Broth (TSB) or Eosin Methylene Blue (EMB) agar at 37 °C for 24 h (h); *G. vaginalis* in NYC III broth at 37 °C in of 5% CO_2_ atmosphere for 24 h; *N. gonhorreae* was grown on Chocolate Enriched agar and *T. vaginalis* on 2154 ATCC Medium at 35 °C for 3–5 days; *C. glabrata* and *C. albicans* were cultivated in Sabouraud Dextrose (SD) broth at 30 °C in aerobiosis.

Each fresh culture of pathogen strain was collected and resuspended in saline solution to obtain a concentration range between 10^7^–10^8^ CFU/mL. The optical densities of the bacterial cultures at 600 nm were measured.

### 4.2. 3D Reconstructed Human Epithelia

Reconstructed human vaginal epithelium (HVE) of 0.5 cm^2^ size (STERLAB, batch n° 2008 VAG01, Vallauris, France) was cultivated for 5 days starting from A431 cell line, reconstituted by airlifted culture on insert polycarbonate filter 0,4 µm in a specific maintenance medium.

3D human reconstructed bladder epithelium (HBE) of 0.5 cm^2^ size (EPISKIN SAS, batch n° 18SMM022, Lyon, France) was formed after 5 days of air-lift culture of immortalized cell line (RT-112) in a chemically defined medium.

On the day of arrival, the HVE and HBE tissues were immediately transferred to a 6-well plate with 1 mL of maintenance medium and placed in incubator at 37 ± 1 °C and 5% CO_2_ for 24 h before the experiments. HVE and HBE batches were tested for the absence of Human Immunodeficiency virus (HIV), Hepatitis B and C, and mycoplasma.

### 4.3. Antimicrobial and Antiadhesive Efficacy on Reconstructed HVE

Two different protocols were applied to assess SynBalance^®^ Femme efficacy: the first for antimicrobial efficacy, and the second for preventive and antiadhesive performance. Each pathogen was tested with both protocols in parallel and each condition was investigated in triplicate.

Antimicrobial efficacy protocol (Protocol A): SynBalance^®^ Femme, at 10^7^ CFU/tissue was applied for 16 h on HVE previously colonized by 30 µL of *C. glabrata*, *N. gonhorreae* or *T. vaginalis* (10^7^ CFU/tissue, contact time of 4 h to induce an infection).

Preventive and antiadhesive performance protocol (Protocol B): SynBalance^®^ Femme, at 10^7^ CFU/tissue, was applied directly on HVE tissues for 16 h and then the infection was induced by 30 µL of each three pathogens (107 CFU/tissue for 4 h of contact).

The total viable bacteria count was performed at the end of the treatment on non-adherent microorganisms (washing), attached microorganisms (tissue homogenates), and microorganism penetrated the epithelium and found in the underlying maintenance medium (medium), according to an internal procedure. After incubation, microorganisms of the washing fraction were obtained by adding 2 mL of Dulbecco’s Phosphate Buffered Saline (DPBS, Biowest, Nuaillé, France) to each tissue. To recollect adherent microorganisms, 2 mL of Tryton X-100 Solution 0.1% (MatTek, Ashland, OH, USA) was added to the tissue that was then homogenized. The tissue maintenance medium was also collected. To perform a cell count of each strain, the suspensions obtained were diluted and plated on selective media. For *T. vaginalis* the count was performed after staining with Trypan Blue solution (Sigma Aldrich, St. Louis, MO, USA), by microscope observation in a Bürker chamber to distinguish viable cells from non-viable ones.

At the end of the procedure, the integrity and viability of the tissues were assessed. The integrity of the epithelium barrier was determined by measuring the transepithelial/transendothelial electrical resistance (TEER) using the MilliCell^®^-ERS-2 Volt-Ohm meter and electrode system (Merck Millipore, Burlington, NJ, USA). Tissue viability was assessed based on the ability of the yellow dye MTT (3-(4,5-dimethylthiazol-2-yl)-2,5-diphenyltetrazolium bromide, thiazolyl blue) to be reduced to purple formazan crystals by metabolically active cells. Results are expressed as a percentage of initial value (baseline).

### 4.4. Antimicrobial and Antiadhesive Efficacy on HBE

The same protocols previously described were applied to HBE tissues, analyzing the strains contained in the SynBalance^®^ Femme against *E. coli*. In particular, each probiotic strain was tested for its putative antimicrobial efficacy (Protocol A) and antimicrobial adhesion efficacy (Protocol B) in parallel and each condition was analyzed in triplicate.

Protocol A: the HBE tissues were exposed to 30 μL of *E. coli* (about 7.60 log_10_ CFU/tissue, OD_600 nm_ = 0.8) for 4 h; then, the infected HBE tissues were treated with 30 μL of probiotic strain suspension (OD_600 nm_ = 0.8) for 16 h to mimic a realistic daily exposure time.

Protocol B: the HBE tissues were pre-treated with 30 μL of probiotic strain suspension (OD_600 nm_ = 0.8) for 16 h; then, the HBE tissues were infected with 30 μL of *E. coli* (OD_600 nm_ = 0.8) for 4 h.

The results obtained on the tissues colonized by *E. coli* and treated with the probiotic suspensions were compared with a positive control, colonized by *E. coli* only, and a negative sterile control.

The total viable bacteria count was performed on apical (non-adherent bacteria) and tissue (adherent bacteria) homogenates fractions, according to an internal procedure. The viable counts on apical and homogenate samples were performed using appropriate 10-fold dilutions in saline solution. A volume of 10 µL of each dilution (as well as the starting suspension) were plated by drop plate method on EMB agar and incubated at 37 °C for 24–48 h, in duplicate. The CFU/mL data were then converted in CFU/cm^2^.

Tissue integrity was assessed at the end of each experiment. TEER was measured before infection, after 4 h of colonization and 16 h of probiotic treatment. To perform the measurement, 500 μL of saline solution was directly applied on the tissues that were then placed into a plate containing 5 mL of saline solution. Three measurements for each tissue were carried out. Experiments were performed on duplicate tissues and results were expressed as mean ± SD.

Treated HBE tissues were collected for ultrastructural analysis by Field Emission Scanning Electron Microscope (FE-SEM) (in simplicate). HBE samples were fixed with 2.5% glutaraldehyde solution in PBS 0.1 mol/L for 24 h. Before the analysis, the samples were washed in 0.1 mol/L sodium cacodylate buffer at pH 7.4 and then carried out in 1% osmium tetraoxide (OsO4) in the same buffer (2 h at room temperature, RT). They were dehydrated in increasing concentration of ethanol and hesamethyldisilazane overnight at RT. Finally, samples were placed on pins, with carbon tablets coated with a layer of gold, using Polaron Equipment limited SEM coating unit E5100 and then transferred to the FE-SEM for viewing and image acquisition.

### 4.5. Co-Aggregation Evaluation

To evaluate a possible co-aggregation of the probiotic strains with vaginal pathogens *G. vaginalis*, *E. coli,* and *C. albicans*, 100 mg of each probiotic strain or SynBalance^®^ Femme were resuspended in a buffer solution at pH 5.5. A concentration of 10^9^ CFU/mL was checked by spectrophotometer determination at OD_600 nm_ in comparison with buffer solution (blank). Similarly, each of the three pathogen strains, grown on agar, was resuspended in saline solution and 108 CFU/mL were spectrophotometrically checked in comparison with saline solution (blank). Each test was prepared by mixing 1:1 the two suspensions (probiotics and pathogens) on a slide and leaving them until the formation of aggregates. A visual check of precipitate formation was performed and the flocculation time (precipitate forming) was recorded in order to define the time range of co-aggregation. Then, each sample was prepared for SEM analysis.

Precipitates were collected after eliminating the excess of diluent and an aliquot was transferred onto a glass support designated for SEM analysis; each precipitate was covered with 10 µL of fixative buffer solution and stored at 2–8 °C. Samples were placed on pins with carbon tabs coated with a layer of gold using Polaron Equipment limited SEM coating unit E5100 and then transferred to the Electronic Microscope: FEI Nova Nano SEM 600 (FEI, Beaverton, USA; Field Emission Gun (FEG)-SEM acquisitions by SE dwell 20 ms HV5 Kv). The magnifications were selected based on the morphology and dimensions of the pathogen and the probiotic strains to highlight the formation of aggregates within the precipitate. SEM acquisition was performed by Service Biotech, Naples, Italy.

## 5. Conclusions

In conclusion, the presented in vitro data confirm that *L. plantarum* PBS067, *L. rhamnosus* LRH020, *B. animalis* subsp. *lactis* BL050, and their combination SynBalance^®^ Femme are able to compete and prevent the development of the most common urogenital pathogens. In particular, the production of antimicrobial substances, the competition in adhesion to the epithelial cells, and the co-aggregation ability could explain their mechanism of action in counteracting pathogens infection. However, further investigations are necessary to confirm these activities against other pathogenic microorganisms involved in urinary tract and vaginal infections.

## Figures and Tables

**Table 1:** In vitro model of antimicrobial efficacy of SynBalance^®^ Femme vs. *C. glabrata* ATCC 15126, *N. gonhorreae* ATCC 43069 or *T. vaginalis* ATCC 30238 on HVE. The amount of SynBalance^®^ Femme and each pathogen was expressed as log_10_ CFU/mL. The epithelium viability was expressed in percentage, while the TEER in Ohm × cm^2^. All the experiments were carried out in triplicate.

Protocol A	SynBalance^®^ Femme	*C. glabrata*	SynBalance^®^ Femme	*N. gonhorreae*	SynBalance^®^ Femme	*T. vaginalis*
Initial count	7.74	7.75	7.47	7.81	7.96	7.49
Non-adhered microrganism (washing)	6.68	7.75	0	0	4.60	0
Adhered microrganism (homogenates)	7.30	5.43	6.10	4.69	6.68	0
Microrganism penetrated into the epithelium (medium)	0	0	0	0	0	0
Epithelium viability (%)	99.0	-	98.7	-	98.7	-
Epithelium integrity (TEER, Ω)	173.3	-	175.4	-	173.8	-

- not performed.

**Table 2:** In vitro model of preventive and antiadhesive efficacy of SynBalance^®^ Femme vs. *C. glabrata* ATCC 15126, *N. gonhorreae* ATCC 43069 or *T. vaginalis* ATCC 30238 on HVE. The amount of SynBalance^®^ Femme or each pathogen was expressed as log_10_ CFU/mL. The epithelium viability was expressed in percentage, while the TEER in Ohm × cm^2^. All the experiments were carried out in triplicate.

Protocol B	SynBalance^®^ Femme	*C. glabrata*	SynBalance^®^ Femme	*N. gonhorreae*	SynBalance^®^ Femme	*T. vaginalis*
Initial count	7.74	7.75	7.47	7.81	7.96	7.49
Non-adhered microrganism (washing)	6.20	6.17	0	0	4.84	2.17
Adhered microrganism (homogenated)	7.83	0	5.95	5.14	3.95	3.48
Microrganism penetrated into epithelium (medium)	0	0	0	0	0	0
Epithelium viability (%)	99.1	-	96.8	-	95.1	-
Epithelium integrity (TEER, Ω)	170.7	-	171.0	-	170.9	-

- not performed.

**Table 3:** Antimicrobial and preventive-antiadhesive efficacy of *B. animalis* subsp. *lactis* BL050, *L. plantarum* PBS067 and *L. rhamnosus* LRH020 vs. *E. coli* ATCC 8739 on HBE. The amount of each strain and the pathogen was expressed as log_10_/CFU/mL.

	Protocol A		Protocol B	
	APICAL log_10_ CFU/mL	HOMOGENATE log_10_ CFU/mL	APICAL log_10_ CFU/mL	HOMOGENATE log_10_ CFU/mL
*E. coli*	7.18	5.90	6.56	5.96
*B. animalis* subsp. *lactis* BL050	0.00	4.38	6.38	4.08
*L. plantarum* PBS067	4.63	0.00	6.63	4.93
*L. rhamnosus* LRH020	5.11	0.00	5.45	0.00

**Table 4:** Trans-Epithelial-Electrical-Resistance (TEER) expressed in Ohm × cm^2^ at baseline (only in the controls), at the end of the colonization with *E. coli*, and at the end of treatment with probiotics (20 h).

	Protocol A		Protocol B	
	Baseline	T = 20 h	Baseline	T = 20 h
Negative control	71.00 ± 0.94	76.25 ± 0.35	71.08 ± 1.53	75.58 ± 0.59
*E. coli*	71.08 ± 1.77	73.83 ± 1.41	71.42 ± 1.06	69.92 ± 3.18
*B. animalis* subsp. *lactis* BL050	-	71.58 ± 4.12	-	68.33 ± 1.65
*L. plantarum* PBS067	-	73.08 ± 2.24	-	70.75 ± 5.30
*L. rhamnosus* LRH020	-	69.25 ± 1.06	-	72.17 ± 1.18

**Table 5:** Results of viable count obtained by the decrease in *E. coli* viability on HBE, expressed as log_10_ variation with respect to the colonized control, for both protocols.

	Protocol A		Protocol B	
Strain Tested	APICAL	HOMOGENATE	APICAL	HOMOGENATE
*B. animalis* supsp. *lactis* BL050	>7 log_10_ (total depletion)	>1 log_10_	No variation	>1 log_10_
*L. plantarum* PBS067	>2 log_10_	>5 log_10_ (total depletion)	No variation	1 log_10_
*L. rhamnosus* LRH020	>1 log_10_	>5 log_10_ (total depletion)	>1 log_10_	>5 log_10_ (total depletion)

**Table 6:** The visual check of precipitate was expressed as cluster quantity (−, +, ++, +++) and the flocculation time was recorded in minutes (min).

Probiotic Strain/Formulation	Pathogen	Precipitate Formation	Flocculation Time
*B. animalis* subsp. *lactis* BL050	*Gardnerella vaginalis*	+	>30 min
	*Escherichia coli*	+	>30 min
	*Candida albicans*	++	15 < min <30
*L. plantarum* PBS067	*Gardnerella vaginalis*	−	>30 min
	*Escherichia coli*	−	>30 min
	*Candida albicans*	−	>30 min
*L. rhamnosus* LRH020	*Gardnerella vaginalis*	+++	<15 min
	*Escherichia coli*	+++	<15 min
	*Candida albicans*	+++	<15 min
SynBalance^®^ Femme	*Gardnerella vaginalis*	+++	<15 min
	*Escherichia coli*	+++	<15 min
	*Candida albicans*	+++	<15 min

## Data Availability

All relevant data are within the manuscript.
